# Response Prediction in Chronic Hepatitis C by Assessment of IP-10 and *IL28B*-Related Single Nucleotide Polymorphisms

**DOI:** 10.1371/journal.pone.0017232

**Published:** 2011-02-24

**Authors:** Martin Lagging, Galia Askarieh, Francesco Negro, Stephanie Bibert, Jonas Söderholm, Johan Westin, Magnus Lindh, Ana Romero, Gabriele Missale, Carlo Ferrari, Avidan U. Neumann, Jean-Michel Pawlotsky, Bart L. Haagmans, Stefan Zeuzem, Pierre-Yves Bochud, Kristoffer Hellstrand, for the DITTO-HCV Study Group

**Affiliations:** 1 Department of Infectious Diseases/Virology, University of Gothenburg, Gothenburg, Sweden; 2 University Hospital of Geneva, Geneva, Switzerland; 3 Infectious Diseases Service, University Hospital and University of Lausanne, Lausanne, Switzerland; 4 Azienda Ospedaliera di Parma, Parma, Italy; 5 Bar-Ilan University, Ramat-Gan, Israel; 6 Hôpital Henri Mondor - Université Paris XII, Créteil, France; 7 Department of Virology, Erasmus MC, Rotterdam, The Netherlands; 8 Department of Medicine I, J. W. Goethe University Hospital, Frankfurt, Germany; Saint Louis University, United States of America

## Abstract

**Background:**

High baseline levels of IP-10 predict a slower first phase decline in HCV RNA and a poor outcome following interferon/ribavirin therapy in patients with chronic hepatitis C. Several recent studies report that single nucleotide polymorphisms (SNPs) adjacent to *IL28B* predict spontaneous resolution of HCV infection and outcome of treatment among HCV genotype 1 infected patients.

**Methods and Findings:**

In the present study, we correlated the occurrence of variants at three such SNPs (*rs12979860*, *rs12980275*, and *rs8099917*) with pretreatment plasma IP-10 and HCV RNA throughout therapy within a phase III treatment trial (HCV-DITTO) involving 253 Caucasian patients. The favorable SNP variants (CC, AA, and TT, respectively) were associated with lower baseline IP-10 (P = 0.02, P = 0.01, P = 0.04) and were less common among HCV genotype 1 infected patients than genotype 2/3 (P<0.0001, P<0.0001, and P = 0.01). Patients carrying favorable SNP genotypes had higher baseline viral load than those carrying unfavorable variants (P = 0.0013, P = 0.029, P = 0.0004 respectively). Among HCV genotype 1 infected carriers of the favorable C, A, or T alleles, IP-10 below 150 pg/mL significantly predicted a more pronounced reduction of HCV RNA from day 0 to 4 (first phase decline), which translated into increased rates of RVR (62%, 53%, and 39%) and SVR (85%, 76%, and 75% respectively) among homozygous carriers with baseline IP-10 below 150 pg/mL. In multivariate analyses of genotype 1-infected patients, baseline IP-10 and C genotype at *rs12979860* independently predicted the first phase viral decline and RVR, which in turn independently predicted SVR.

**Conclusions:**

Concomitant assessment of pretreatment IP-10 and *IL28B*-related SNPs augments the prediction of the first phase decline in HCV RNA, RVR, and final therapeutic outcome.

## Introduction

Hepatitis C virus (HCV) infects 170 million people worldwide [1] and is a leading cause of chronic hepatitis, cirrhosis, and hepatocellular carcinoma [2]. Treatment with pegylated interferon-α (peg-IFN) and ribavirin results in a sustained viral response (SVR) in approximately 50% of patients infected with HCV of genotype 1 and 80% of those with HCV genotypes 2 or 3 [3], [4], [5]. Recently, several genome-wide association studies have revealed that single nucleotide polymorphisms (SNPs) in the *19q13* region, in close proximity to three genes (*IL28A*, *IL28B*, and *IL29*) encoding cytokines of the IFN-λ (i.e. type III IFN) family, predict spontaneous clearance of HCV infection [6], [7] as well as SVR following peg-IFN/ribavirin therapy among patients infected with HCV genotype 1 [6], [8], [9], [10]. Three of these SNPs are reportedly highly predictive of a favorable treatment response among HCV genotype 1 infected patients: *rs12979860* [7], [8], *rs12980275* [10], and *rs8099917* [6], [9], [10], with a strong linkage disequilibrium noted between *rs12979860* and *rs8099917* [6]. Aside from differences in the assays utilized, SVR was the defined endpoint in the genome-wide association study (GWAS) identifying *rs12979860* [8], whereas null responsiveness to interferon was used in the analysis that endorsed *rs12980275* and *rs8099917* [10].

The C allele at *rs12979860* also is associated with higher baseline viral load [8], [11], which otherwise is an established negative predictor of response to peg-IFN/ribavirin therapy [3], [4], [5]. Similarly counterintuitive is the report that a C allele at *rs12979860* is more common in Caucasians with HCV genotype 2 and 3 infection than in genotype 1 infected or in HCV uninfected individuals [11], [12]. In addition, two studies reported that homozygous carriage of GG at *rs8099917* was associated with slightly lower PBMC mRNA expression of *IL28* in 49 and 20 individuals, respectively [9], [10], although another study reported no difference in *IL28B* mRNA expression when stratified regarding *rs12979860* genotype in PBMC from 80 individuals [8]. In line with this latter finding, G allele carriage at *rs8099917* has been reported to be associated with elevated intrahepatic mRNA expression of a panel of 37 interferon-stimulated genes (ISGs) but not *IL28B* in 91 HCV infected patients [13].

Interferon-gamma inducible protein 10 kDa (IP-10 or CXCL10) is a chemotactic CXC chemokine of 77 amino acids in its mature form [14], [15]. IP-10 targets the CXCR3 receptor but, unlike other CXC chemokines, lacks chemotactic activity for neutrophils and instead attracts T lymphocytes, NK cells, and monocytes to sites of infection [15], [16], [17]. IP-10 is produced by a variety of cells, including hepatocytes, and levels of IP-10 at onset of therapy are reportedly elevated in patients infected with HCV of genotypes 1 or 4 who do not achieve SVR [18]. In difficult-to-treat genotype 1 infected HCV patients, cut-off levels of IP-10 in plasma of 150 pg/mL (approximately equal to 2 standard deviations above the mean IP-10 level of HCV seronegative blood donors) and 600 pg/mL have yielded positive and negative predictive values for SVR of 71% and 100%, respectively [19]. IP-10 in plasma is mirrored by intrahepatic IP-10 mRNA, and strongly predicts the HCV RNA decline during the first days (“first phase decline”) during interferon/ribavirin therapy for all HCV genotypes [20].

The impact of IP-10 on the elimination of HCV RNA during therapy in the setting of *IL28B* genetic variants is not known. We therefore assessed plasma IP-10 in relation to genetic variants at three major *IL28B* SNPs (*rs12979860*, *rs12980275*, and *rs8099917*) in patients chronically infected with HCV genotypes 1-4. Our results demonstrate a significant association between *IL28B* genetic variants and IP-10 in plasma and imply that combined assessment of these predictive factors may improve prognostication of the rate of first phase elimination of HCV RNA, as well as achieving a rapid virological response (RVR) and SVR.

## Methods

### Ethical Aspects

The treatment study conformed to the guidelines of the 1975 Declaration of Helsinki and was approved by ethical committees at each center (Medicinska fakultetens forskningsetikkommitté, Göteborgs Universitet, Gothenburg, Sweden, CPP-Ile-de-France IX, CHU Henri Mondor, Creteil, France, Comitato Etico Indipendente (IRB/IEC) dell'Azienda Ospedaliera di Parma, Parma, Italy, Comite d'Ethique du department de Medicine, Hopitaux Universitaires de Genève, Geneva, Switzerland, the Helsinki committee of the Kaplan Medical Center, Rehovot, Israel, the Ethics Committee of Hospital General Vall d'Hebron, Barcelona, Spain, the Ethics Committee of Aristotle University of Thessaloniki, Thessaloniki, Greece, the Ethics Committee of Klinikum der Johann Wolfgang Goethe-Universitat, Frankfurt, Germany, the Ethics Committee of University Hospital Rotterdam Dijkzigt, Rotterdam, Netherlands). Written informed consent was obtained from each patient included in this study.

### Patients

Between February 2001 and November 2003, 270 patients (180 men and 90 women) were recruited in a phase III, open-label, randomized, multicenter trial conducted by the DITTO-HCV study group at 9 centers in France, Germany, Greece, Israel, Italy, Netherlands, Spain, Sweden, and Switzerland, as previously reported [21]. All patients were adults, had compensated liver disease, were treatment naïve for hepatitis C, and fulfilled the following inclusion criteria: a positive test for anti-HCV antibody, an HCV RNA level greater than 1000 IU/mL, and two serum alanine aminotransferase values above the upper limit of normal within 6 months of treatment initiation. Two hundred and fifty three Caucasian patients had samples available for *IL28B* analysis (baseline characteristics shown in Table 1), and 252 of these patients had pretreatment plasma available for evaluation of IP-10.

**Table 1:** Baseline Characteristics with Patients Grouped According to *IL28B* Genetic Variations.

NCBI dbSNP ID	*rs12979860*				*rs12980275*				*rs8099917*			
	CC	CT	TT	P	AA	AG	GG	P	TT	TG	GG	P
	(n = 93)	(n = 123)	(n = 37)		(n = 101)	(n = 115)	(n = 37)		(n = 153)	(n = 90)	(n = 10)	
Age (years)^a^	41.6 (10.1)	41.9 (9.5)	41.9 (11.4)	0.85^c^	41.7 (10.1)	41.8 (10.3)	41.0 (11.5)	0.93^c^	42.1 (9.2)	40.5 (11.1)	45.0 (12.5)	0.23^c^
Gender (Male/Female)^b^	64/29	77/46	28/8	0.18^d^	65/36	78/37	26/10	0.62^d^	102/51	60/29	7/3	0.97^d^
BMI (kg/m^2^)^a^	25.1 (3.6)	25.0 (3.5)	25.0 (3.5)	0.91^c^	24.9 (3.7)	25.3 (3.7)	25.1 (3.3)	0.69^c^	25.1 (3.6)	25.1 (3.5)	26.3 (4.1)	0.55^c^
HCV-RNA level (log_10_ IU/mL)^a^	6.3 (0.8)	6.1 (0.7)	5.9 (0.8)	0.0013^c^	6.3 (0.8)	6.1 (0.8)	6.0 (0.6)	0.029^c^	6.3 (0.8)	5.9 (0.7)	5.8 (0.8)	0.0004^c^
HCV Genotype (1/2/3/4)^b^	44/13/33/3	96/7/15/5	30/3/1/3	<0.0001^d^	52/13/33/3	88/7/15/5	30/3/1/3	<0.0001^d^	97/15/38/3	66/7/11/6	7/1/0/2	0.01^d^
Fibrosis Stage (Ishak 0/1/2/3/4/5/6)^b^	3/18/27/11/5/12/5	7/35/27/17/7/6/6	1/8/11/2/3/2/3	0.5^d^	4/22/28/12/6/12/4	6/31/25/16/6/6/7	1/8/12/2/3/2/3	0.71^d^	6/36/46/22/7/14/5	5/22/17/8/8/5/7	0/3/2/0/0/1/2	0.18^d^
Steatosis Grade (0/1/2/3)^b^	31/33/12/5	54/35/14/1	13/13/3/1	0.35^d^	35/35/13/5	48/33/13/2	15/13/3/0	0.54^d^	60/53/16/7	35/24/12/0	3/4/1/0	0.43^d^

a Data presented as mean (SD) or

b n.

c Kruskal-Wallis Test.

d Chi Squared test.

### Treatment

All patients in the DITTO-HCV trial were treated for 6 weeks with 180 µg pegylated interferon-α2a sc once weekly (Pegasys, F. Hoffmann-LaRoche, Basel, Switzerland) and ribavirin orally twice daily (Copegus, F. Hoffmann-LaRoche) at a total daily dose of 1,000 mg for patients weighing less than 75 kg and 1,200 mg daily for above 75 kg. Thereafter, patients were randomized 1∶1 based on their viral kinetic classification to receive individualized therapy or to continue on standard combination therapy for a total of 48 weeks. There were no major differences in treatment outcome for patients receiving individualized or standard therapy [21].

### HCV Genotyping

Genotyping of HCV was performed using INNO-LiPA HCV II (Innogenetics N.V., Ghent, Belgium).

### HCV RNA Quantification

HCV RNA was determined by RT-PCR using Cobas Amplicor HCV Monitor version 2.0 (Roche Diagnostics, Branchburg, NJ), and quantified on days 0, 1, 4, 7, 8, 15, 22, 29, at end of treatment, and 24 months after the completion of treatment.

### Classification of Treatment Outcome

Patients were classified as having achieved RVR if HCV RNA was undetectable (<50 IU/mL) in plasma on treatment day 29, and were classified as having SVR if HCV RNA was undetectable in plasma 24 weeks after the completion of therapy.

### Liver Biopsies

Liver biopsies were obtained from patients in the DITTO-HCV trial within 12 months prior to inclusion in the study, and liver biopsy samples were processed for both histological evaluation (≥1.5 cm) and for RNA analysis (≥1 cm). Only biopsies with a length exceeding 1.5 cm and containing more than 6 portal tracts were evaluated. In total, liver biopsies from 228 infected patients in the DITTO-HCV trial were retrieved and evaluated. For each biopsy, a hematoxylin-eosin stain and a Sirius Red stain were centrally staged and graded by two independent observers experienced in pseudo-numerical scoring of liver biopsies in a blinded fashion according to the Ishak protocol [22]. Equivocal issues were debated after the independent scores were noted, and a consensus score was obtained. In addition, steatosis was graded as follows: absent  = 0, less than 30% of hepatocytes involved  = 1, 30–70% of hepatocytes involved  = 2, and more than 70% of hepatocytes involved  = 3 [23].

### IP-10 Quantification in Plasma

Quantification of IP-10 was performed using Quantikine (R&D SYSTEMS Minneapolis, MN), a solid-phase ELISA, on plasma samples obtained during the week prior to the start of therapy. All samples were stored at –70°C until assayed.

### DNA extraction

DNA from peripheral blood mononuclear cells was isolated using the QIAamp DNA mini kit (Qiagen) and quantitated on the NanoDrop 1000 spectrophotometer (Thermo Fischer Scientific).

### IL28-B genotyping

DNA samples from patients and controls were genotyped for the *IL28B rs8099917*, *rs12979860* and *rs12980275* polymorphism with TaqMan SNP genotyping assays (Applied Biosystems Inc, Foster City, CA), using the ABI 7500 Fast real time thermocycler, according to manufacturers recommended protocols. TaqMan probes and primers were designed and synthesized by Applied Biosystems Inc. Automated allele calling was performed using SDS software from Applied Biosystems Inc. Positive and negative controls were used in each genotyping assay. The primers and probes utilized were:

1. NCBI dbSNP ID ***rs8099917*** Applied Biosystems (AB) reference: C_11710096_10
2. NCBI dbSNP ID ***rs12979860*** **Forward primer:** TGTACTGAACCAGGGAGCTC, **Reverse primer:** GCGCGGAGTGCAATTCAAC, **Vic probe:** TGGTTCGCGCCTTC, **Fam probe:** TGGTTCACGCCTTC
3. NCBI dbSNP ID ***rs12980275*** **Forward primer:** GTGCTGAGAGAAGTCAAATTCC, **Reverse primer:** CCGCTACCCGGCAAATATT, **Vic probe:** AGACACGTCTGTTTCTA, **Fam probe:** ACACGTCCGTTTCTA

### Statistical Methods

Individual characteristics between groups were evaluated using the Wilcoxon-Mann-Whitney U-test, Kruskal-Wallis test and Chi squared (χ^2^) test, and Spearman's rank correlation coefficient *r_s_* test was utilized to evaluate relationships between variables. All abovementioned statistical analyses were performed using StatView for Macintosh (Version 5.0, SAS Institute Inc., Cary, NC, USA). After univariate analyses, multivariate analyses were performed among the HCV genotype 1 infected patients using Stata (version 9.1, StataCorp, College Station, TX, USA). For multivariate models, all variables associated with the endpoint (P<0.2) were entered, with age and gender forced into the model. Because of strong linkage disequilibrium noted between *rs12979860* and *rs12980275* (*R^2^* = 0.86 assuming Hardy-Weinberg equilibrium, as compared to *R^2^* = 0.42 for *rs12979860* and *rs8099917* as well as *R^2^* = 0.41 for *rs12980275* and *rs8099917*) and because *rs8099917* was not significantly associated with SVR, only SNP variants for *rs12979860* were included in the multivariate analyses. SNPs were tested using three models assuming one of the following modes of inheritance: dominant (comparing presence of one or two copies of the minor allele versus none), recessive (comparing presence of two copies of the minor allele versus none or one copy), and additive (none, one or two copies of the minor allele were coded 0, 1 and 2, respectively, assuming greater effect with increased copy number of the minor allele). Linkage disequilibrium was calculated using the pwld software implemented in Stata. All reported p-values are two-sided, and p-values <0.05 were considered significant.

## Results

**Figure 1:**
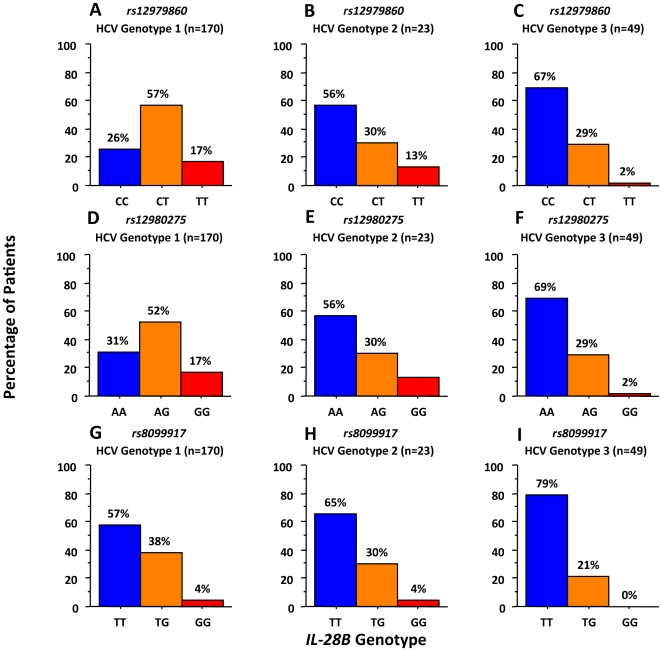
Frequency distribution of *IL28B* variants in relation to HCV genotypes 1-3.

A strong association was noted between the distribution of HCV genotypes and *IL28B* SNP variants (P<0.0001 for *rs12979860* and *rs12980275*, and P = 0.01 for *rs8099917*, Chi squared test; Table 1) with the favorable CC at *rs12979860*, AA at *rs12980275*, and TT at *rs8099917* being significantly more common in patients with HCV genotype 2 or 3 infection than genotype 1 (Figure 1). The 11 patients infected with HCV genotype 4 had a similar distribution of *IL28B* variants as patients infected with genotype 1. Patients who were homozygous for the favorable SNP genotypes had higher baseline viral load (mean 6.3 log_10_ IU/mL; Table 1); heterozygous patients had intermediate (mean 6.1 log_10_ IU/mL for *rs12979860* and *rs12980275*, and 5.9 log_10_ IU/mL for TG at *rs8099917*); and patients who were homozygous for the risk alleles had lower (mean 5.9 log_10_ IU/mL for TT at *rs12979860*, 6.0 log_10_ IU/mL for GG at *rs12980275*, and 5.8 log_10_ IU/mL for GG at *rs8099917*, P = 0.0013, P = 0.029, and P = 0.0004 respectively; Table 1).

**Figure 2:**
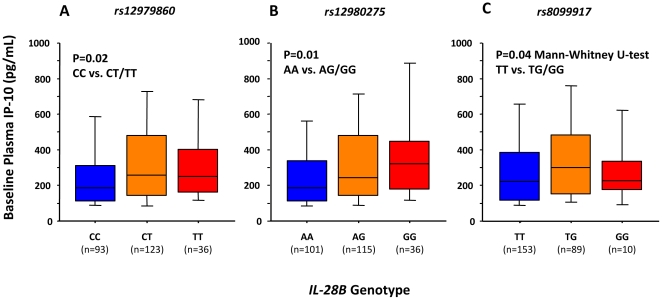
Tenth, 25^th^, 50^th^, 75^th^, and 90^th^ percentiles of pretreatment IP-10 in relation to *IL28B* variants.

Significantly lower baseline plasma IP-10 levels were observed in homozygous carriers of the favorable CC at *rs12979860* (median 189 vs. 258 pg/mL, P = 0.02, Mann-Whitney U-test; Figure 2), AA at *rs12980275* (median 189 vs. 258 pg/mL, P = 0.01), and TT at *rs8099917* (median 224 vs. 288 pg/mL, P = 0.04), as compared with patients carrying one or two copies of the risk alleles.

**Figure 3:**
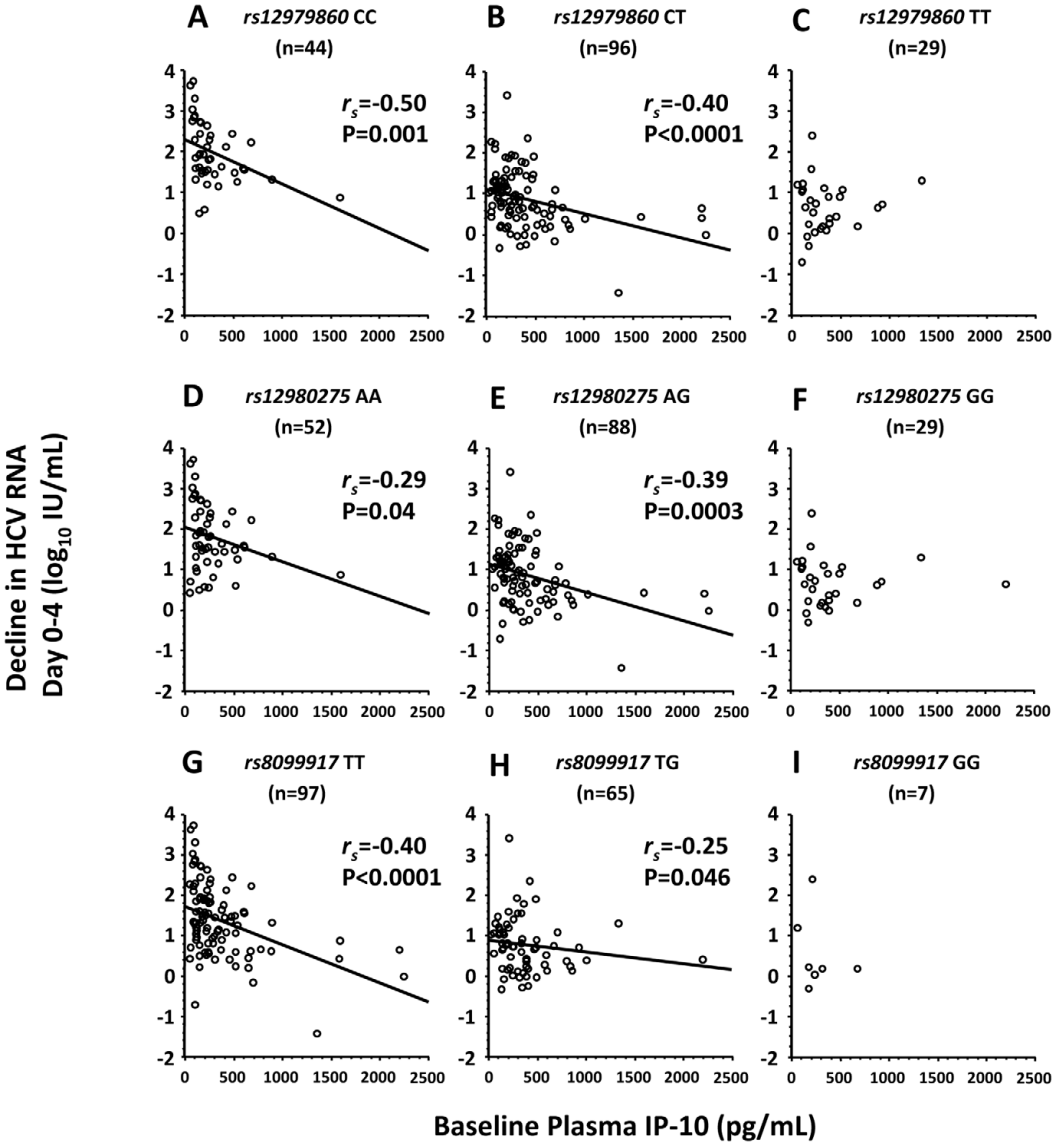
Correlation between IP-10 and first phase decline in *IL28B* variants among HCV genotype 1 patients.

**Figure 4:**
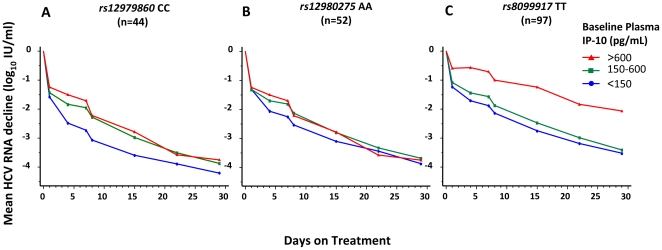
Mean HCV RNA reduction according to IP-10 in HCV genotype 1 with favorable *IL28B* genotype.

Among genotype 1 infected patients, homozygous carriers of the three favorable *IL28B* alleles had significantly more pronounced first phase viral decline, as reflected by the reduction of HCV RNA from treatment day 0 to 4, when compared with patients carrying the risk alleles (mean 2.0, 0.9 and 0.6 log_10_ IU/mL for *rs12979860* CC, CT and TT, 1.8, 0.9 and 0.7 log_10_ IU/mL for *rs12980275* AA, AG and TT, and 1.4, 0.8 and 0.6 for *rs8099917* TT, TG and GG respectively, P<0.0001 for all 3 SNPs; Kruskal-Wallis test). Among homozygous or heterozygous carriers of the favorable alleles, IP-10 was highly significantly associated with the first phase reduction of HCV RNA (*r_s_* = −0.50, P = 0.001 and *r_s_* = −0.40, P<0.0001 for *rs12979860* CC and CT, *r_s_* = −0.29, P = 0.04 and *r_s_* = −0.39, P = 0.0003 for *rs12980275* AA and AG, and *r_s_* = −0.40, P<0.0001 and *r_s_* = −0.25, P = 0.046 for *rs8099917* TT and TG respectively; Figure 3). This association was also significant for the maximum decline in HCV RNA from day 0 to 4 as well as the decline from day 0 to 1, thus emphasizing an association with the first phase decline in HCV RNA, and translated into a more rapid reduction of HCV RNA during the first 4 weeks of therapy among patients with lower baseline IP-10, and a slower decline among those with higher (Figure 4). Similarly, among genotype 2/3 infected patients, CC carriers of *rs12979860* had significantly more pronounced first phase viral decline, as reflected by the reduction of HCV RNA from treatment day 0 to 4, when compared with patients carrying the risk allele (mean 2.7, 2.1 and 1.6 log_10_ IU/mL for CC, CT and TT, P = 0.04; Kruskal-Wallis test) and IP-10 was significantly correlated with the reduction in HCV RNA from day 0 to 4 in the 22 HCV genotype 2/3 infected patients with *rs12979860* CT (*r_s_* = −0.45, P = 0.04). In contrast to the first phase reduction in HCV RNA, none of the *IL28B*-related SNPs or baseline IP-10 predicted the second phase decline after stratification for the first phase decline.

Both *rs12979860* (P = 0.006) and *rs12980275* (P = 0.007) were significantly predictive of RVR, i.e. undetectable HCV RNA at week 4, whereas the allelic distribution of *rs8099917* was not (P = 0.18). Similarly, baseline plasma IP-10 was lower in patients achieving RVR than in those who did not (median 222 pg/mL vs. 401 pg/mL, P = 0.008; Mann-Whitney U-test), and, among homozygous carriers of the *rs12979860* and *rs8099917* alleles, a lower baseline IP-10 significantly predicted RVR (Table 2). Interestingly none of the patients with baseline IP-10 above 600 pg/mL achieved RVR regardless of *IL28B* genotype.

**Table 2:** The impact of *IL28B* genetic variations and baseline plasma IP-10 on the likelihood of achieving RVR among patients infected with HCV genotype 1.

NCBI dbSNP ID	<150 pg/mL	150–600 pg/mL	>600 pg/mL	P	Total	P^a^
*rs12979860*						
CC	8/13 (62%)	8/25 (32%)	0/4 (0%)	0.05	16/42 (38%)	0.006
CT	4/22 (18%)	11/55 (20%)	0/16 (0%)	0.15	15/93 (16%)	
TT	0/6 (0%)	3/17 (18%)	0/3 (0%)	0.41	3/27 (11%)	
*rs12980275*						
AA	9/17 (53%)	9/29 (31%)	0/4 (0%)	0.10	18/50 (36%)	0.007
AG	3/19 (16%)	10/51 (20%)	0/15 (0%)	0.18	13/85 (15%)	
GG	0/5 (0%)	3/17 (18%)	0/4 (0%)	0.41	3/27 (11%)	
*rs8099917*						
TT	11/28 (39%)	13/53 (25%)	0/14 (0%)	0.02	24/95 (25%)	0.18
TG	1/12 (8%)	9/40 (22%)	0/8 (0%)	0.20	10/61 (16%)	
GG	0/1 (0%)	0/4 (0%)	0/1 (0%)		0/6 (0%)	
Total	12/41 (29%)	22/97 (23%)	0/23 (0%)	0.02		

All P-values using Chi squared test.

a P-values for SNP genotypes and frequency of RVR irrespective of baseline IP-10.

**Figure 5:**
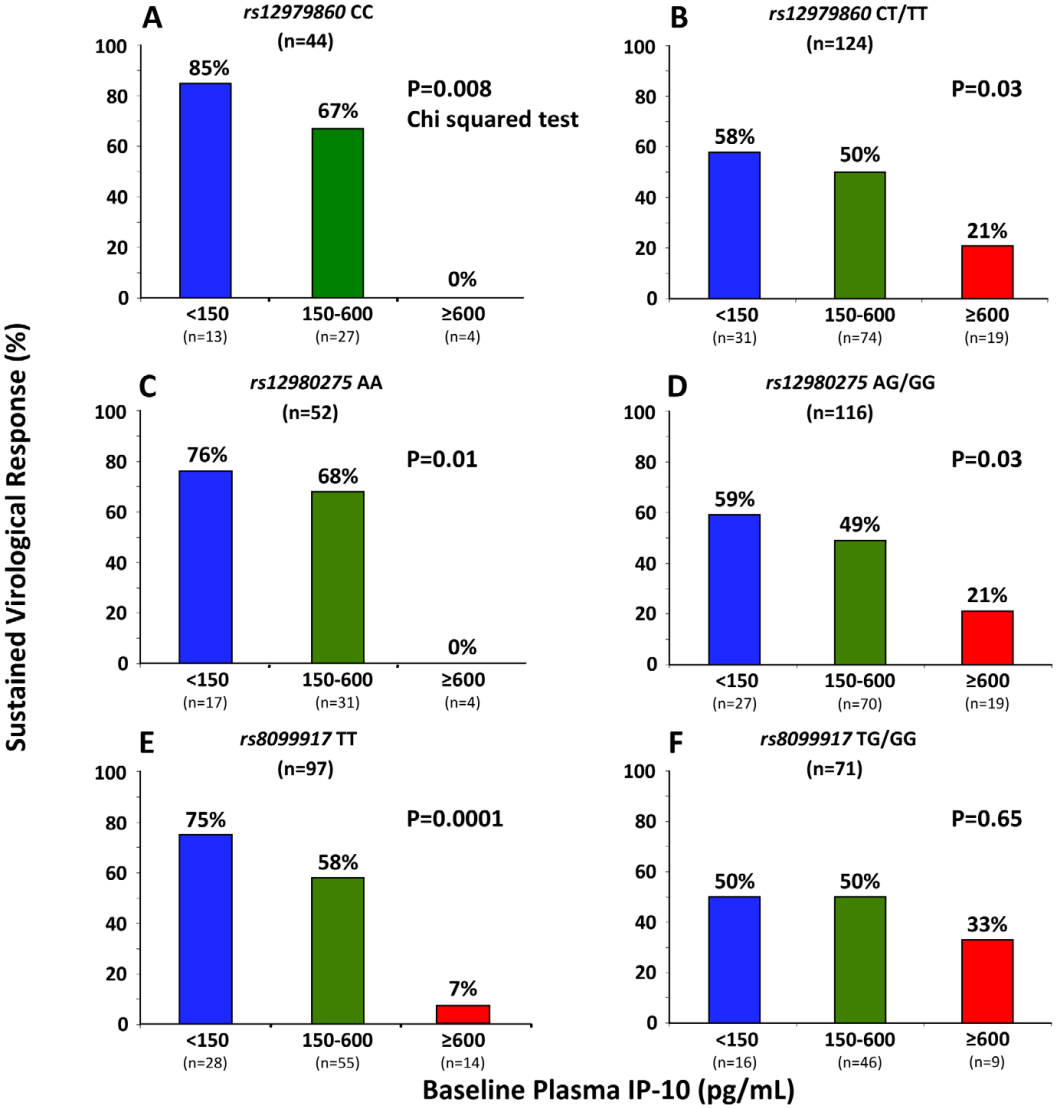
SVR rates in HCV genotype 1 according to *IL28B* variants and baseline IP-10.

The distribution of variants at SNP *rs12979860* and *rs12980275* impacted significantly on SVR among HCV genotype 1 infected patients (66% vs. 48% for *rs12979860* CC and CT/TT, P = 0.04; 65% vs. 47% for *rs12980275* AA and AG/GG, P = 0.03; Chi squared test), but not among variants of *rs8099917*, although a non-significant trend towards lower SVR was noted among the 7 homozygous GG carriers as compared to 162 carriers of TT or TG (29% vs. 54%, P = 0.2). Again, IP-10 significantly augmented the prediction of SVR among genotype 1 infected for all three *IL28B* SNPs with the exception of G allele carriage at *rs8099917* (Figure 5). In patients with baseline IP-10 below 150 pg/mL who were homozygous for favorable SNP genotypes, SVR was achieved in 85%, 75%, and 75% for *rs12979860* CC, *rs12980275* AA, and *rs8099917* TT, respectively. Notably, achieving RVR was slightly more predictive of achieving SVR (91%; Table 3) than combinations of *IL28B* and baseline IP-10. This remained the case even when the analysis was restricted to the genotype 1 infected patients who had no dose reductions, i.e. per-protocol analysis (92% vs. 83% for RVR and *rs12979860* CC and baseline IP-10 below 150 pg/mL respectively; n = 126). In contrast to genotype 1, neither *IL28B* genotype distribution nor baseline IP-10 levels predicted SVR among the 71 HCV genotype 2/3 infected patients.

**Table 3:** Sensitivity, specificity, positive and negative predictive values of the likelihood achieving SVR among patients infected with HCV genotype 1 (n = 170).

	PPV	NPV	Sensitivity	Specificity
*rs12979860* CC (n = 44)	66%	52%	32%	81%
Baseline plasma IP-10	66%	52%	33%	81%
<150 pg/mL (n = 44)				
CC at *rs12979860* **or**	63%	56%	53%	65%
IP-10<150 pg/mL (n = 75)				
CC at *rs12979860* **and**	85%	50%	12%	98%
IP-10<150 pg/mL (n = 13)				
RVR (n = 34)	91%	59%	37%	96%

In multivariate analyses, both IP-10 and C genotype at *rs12979860* were independent predictors of the first phase decline in HCV RNA (P = 0.009 and P<0.0001 respectively) as well as RVR (P = 0.048 and P = 0.016 respectively), and RVR was in turn the only independent predictor of SVR (P = 0.001). RVR remained the only predictor of SVR when the analysis was restricted to the fully compliant patients (P = 0.005) reiterating the importance of monitoring on-treatment response.

## Discussion

In spite of the pending introduction of direct antiviral agents (DAA) in routine clinical practice, interferon-α and ribavirin are likely to retain pivotal roles in the management of chronic HCV infection, and thus predicting responsiveness to interferon/ribavirin-based therapies will remain important. In this setting, the main finding in the present study was that pretreatment plasma levels of IP-10 increased the level of prediction of the first phase decline in HCV RNA among patients carrying *IL28B* SNP variants, which translated into improved prediction of RVR and SVR. Considering the high SVR rates among HCV genotype 1 infected homozygous carriers of CC at *rs12979860*, AA at *rs12980275*, or TT at *rs8099917* with baseline IP-10 levels below 150 pg/mL (85%, 76%, and 75% respectively), these patients, although few in number, should be encouraged to initiate therapy and may be candidates for shortened duration of therapy in line with current treatment guidelines considering the high likelihood of achieving RVR [24], [25]. Additionally, these patients may be suitable for initial inclusion in pending trials with interferon-free DAA regimes, because of the favorable odds of successful salvage therapy with interferon in the event of development of resistance towards these new therapeutic agents.

The finding that *IL28B* variants primarily impact on the first phase decline of HCV RNA [26], as previously reported for IP-10 [20], suggests that both *IL28B* variants and IP-10 are linked to the antiviral effectiveness of peg-IFN and ribavirin in the blocking of the production or release of infectious virions rather than on the clearance of HCV infected cells. The risk alleles, T at *rs12979860*, or G at *rs12980275* and *rs8099917*, were found to be significantly associated with modest elevations of baseline IP-10 levels. This finding is in line with the recent report by Honda et al. that G at *rs8099917* entails higher intrahepatic expression of a panel of 37 representative ISGs not including IP-10 [13], which is typically observed in patients who respond less favorably to treatment [27]. The elevated baseline induction of ISGs among risk allele carriers may explain why these alleles are associated with a lower viral load observed in our study among patients with chronic HCV infection, corroborating previous reports [8], [11].

Our finding that homozygous CC at *rs12979860*, AA at *rs12980275*, and TT at *rs8099917* were significantly more common in the setting of HCV genotype 2 or 3 infection than 1 in a population consisting only of Caucasian patients confirms and extends the findings reported by McCarthy et al. [11]. Indeed, the proportion of CC at *rs12979860* among HCV genotype 2 and 3 infected patients (56% and 67%, respectively) in our study is higher than the reported prevalence in HCV uninfected Caucasians (∼40%), suggesting that this SNP genotype entails a higher likelihood of development of chronic infection following exposure to HCV genotype 2 or 3. This observation, however, may be considered counter-intuitive since homozygous carriage of CC at *rs12979860* was associated with a significantly greater first phase decline of HCV RNA even in genotype 2/3 infected patients. A possible hypothesis for these seemingly contradictory findings is that carriage of the favorable allele C at *rs12979860*, A at *rs12980275* and T at *rs8099917*, being associated with a slightly diminished baseline activation of ISGs, is beneficial for clearance of all HCV genotypes as reflected by the association with a greater first phase decline in HCV RNA during therapy irrespective of HCV genotype, but more advantageous for genotype 1 in comparison to 2/3. In the event of continuous re-exposure to a variety of HCV genotypes following a possible initial spontaneous clearance of HCV, as is often the case among intravenous drug users in addition to the lack of a lasting protective immune response [28], this skewness will exert selective pressure and over time lead to an under-representation of these favorable alleles among genotype 1 patients and an over-representation among 2/3 patients as compared to the non-infected population. This would be the case even if carriage of this SNP genotype were slightly beneficial in the event of a single point exposure to HCV genotype 2 or 3. Supportive of this concept is the non-significant trend towards lower frequency of homozygous CC at *rs12979860* (30%) observed among 27 HCV genotype 2/3 patients infected through exposure to contaminated blood products as compared to 322 genotype 2/3 patients with other routes of infection in another study (NORDynamIC trial) [29]. Similarly, in an elderly population in southern Italy predominantly infected with HCV genotype 2 likely secondary to past iatrogenic exposure, a non-significant trend towards a lower proportion of CC at *rs12979860* was noted among genotype 2/3 infected patients than among non-infected controls (37% vs. 42%) [30].

In conclusion, baseline plasma IP-10 is significantly associated with *IL28B*-related SNPs, and augments the level of predictiveness of the first phase decline in HCV RNA, RVR, and final treatment outcome. Therefore, pre-treatment screening of *IL28B* genetic variants, together with measurement of IP-10 in plasma, may provide useful prognostic information prior to initiating antiviral therapy for HCV.
